# Supplementary material for: Excavation of Genes Responsive to Brassinosteroids by Transcriptome Sequencing in Adiantum flabellulatum Gametophytes
Source: Genes (Basel). 2022 Jun 14;13(6):1061. doi: 10.3390/genes13061061 (PMC9222622; doi:10.3390/genes13061061)
Supplement: Supplementary file 1 [file genes-13-01061-s001.zip › Supplementary Figures.pdf]

Supplementary Figures

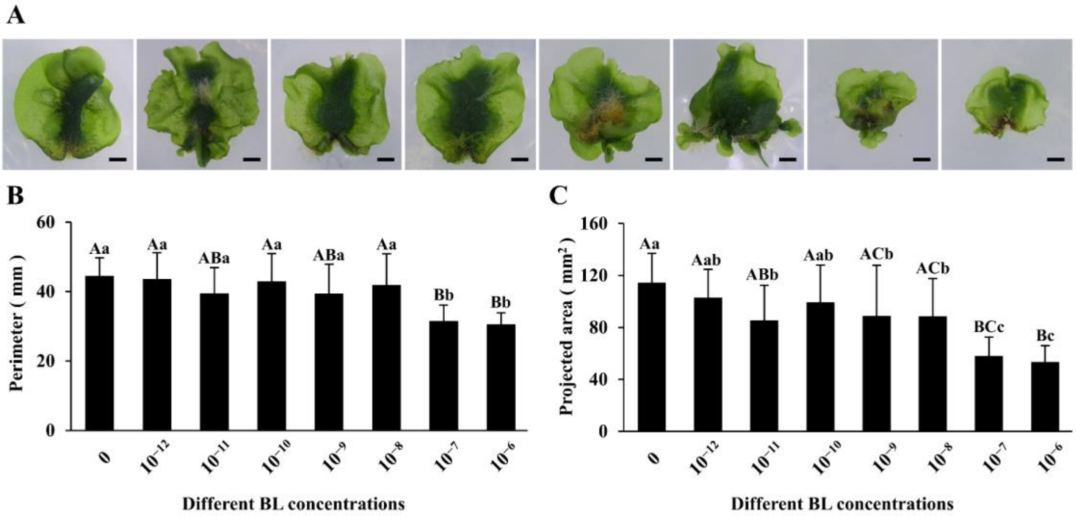

Supplementary Figure S1. *A. flabellulatum* gametophyte growth response to different BL concentrations. **A–C**: *A. flabellulatum* gametophytes cultured for 60 d in 0 M, 10^{-12} M, 10^{-11} M, 10^{-10} M, 10^{-9} M, 10^{-8} M, 10^{-7} M and 10^{-6} M BL respectively (**A**), as well as their perimeters (**B**) and projected areas (**C**). Different lowercase letters or capital letters in different groups indicated statistically significant differences ($p < 0.05$) or statistically extremely significant differences ($p < 0.01$).

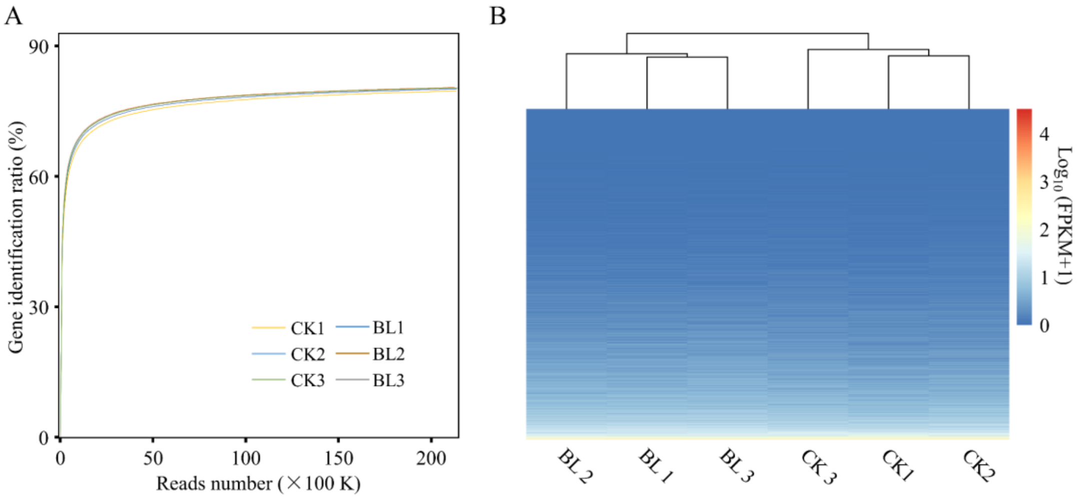

Supplementary Figure S2. Sequencing saturation analysis and cluster analysis of 6 samples. **A**: Sequencing saturation curves of 6 samples; **B**: Cluster analysis among 6 samples of 2 treatments based on the gene expression data. The result showed that 3 biological replicates of each treatment were clustered into one branch.

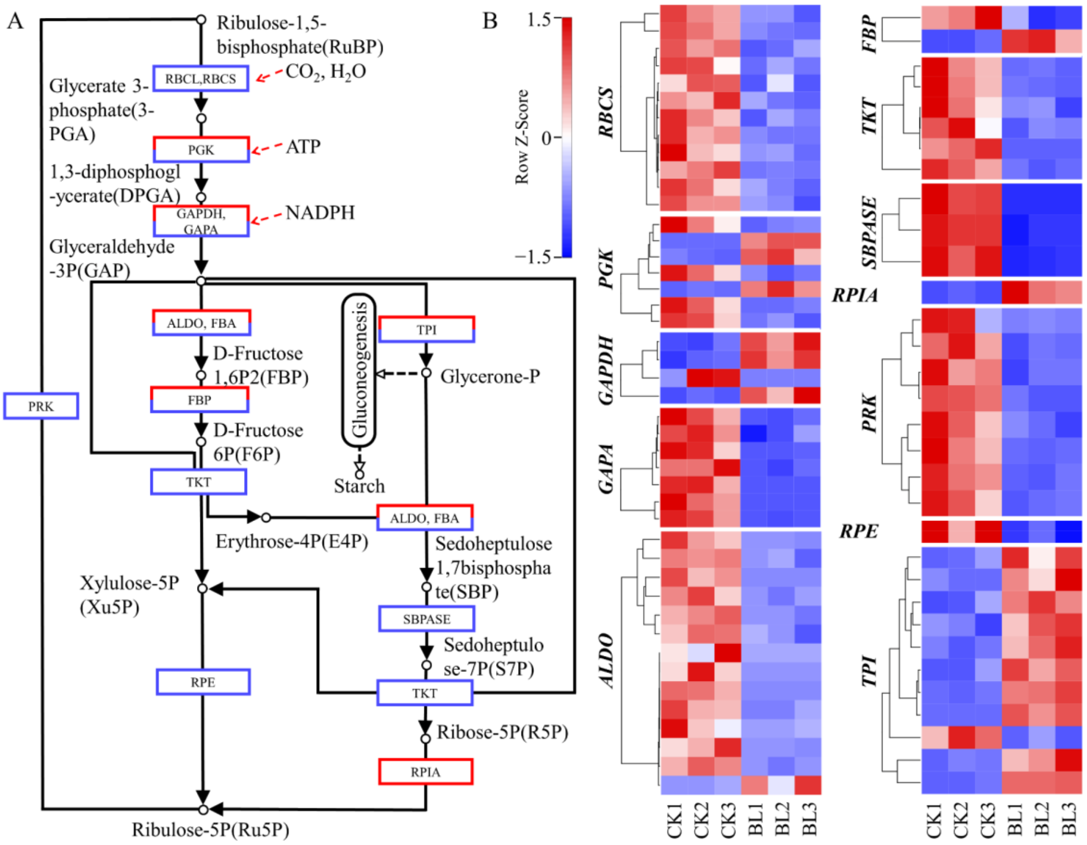

Supplementary Figure S3. Most of the DEGs in Calvin cycle were down-regulated in BL treatment. **A**: Calvin cycle (https://www.kegg.jp/entry/map00710 (accessed on 24 April 2022)), the red and blue square frames indicated all DEGs in the step were up-regulated and down-regulated in BL treatment, respectively (the same below); **B**: Expression heat maps of the DEGs in Calvin cycle in the 6 samples.

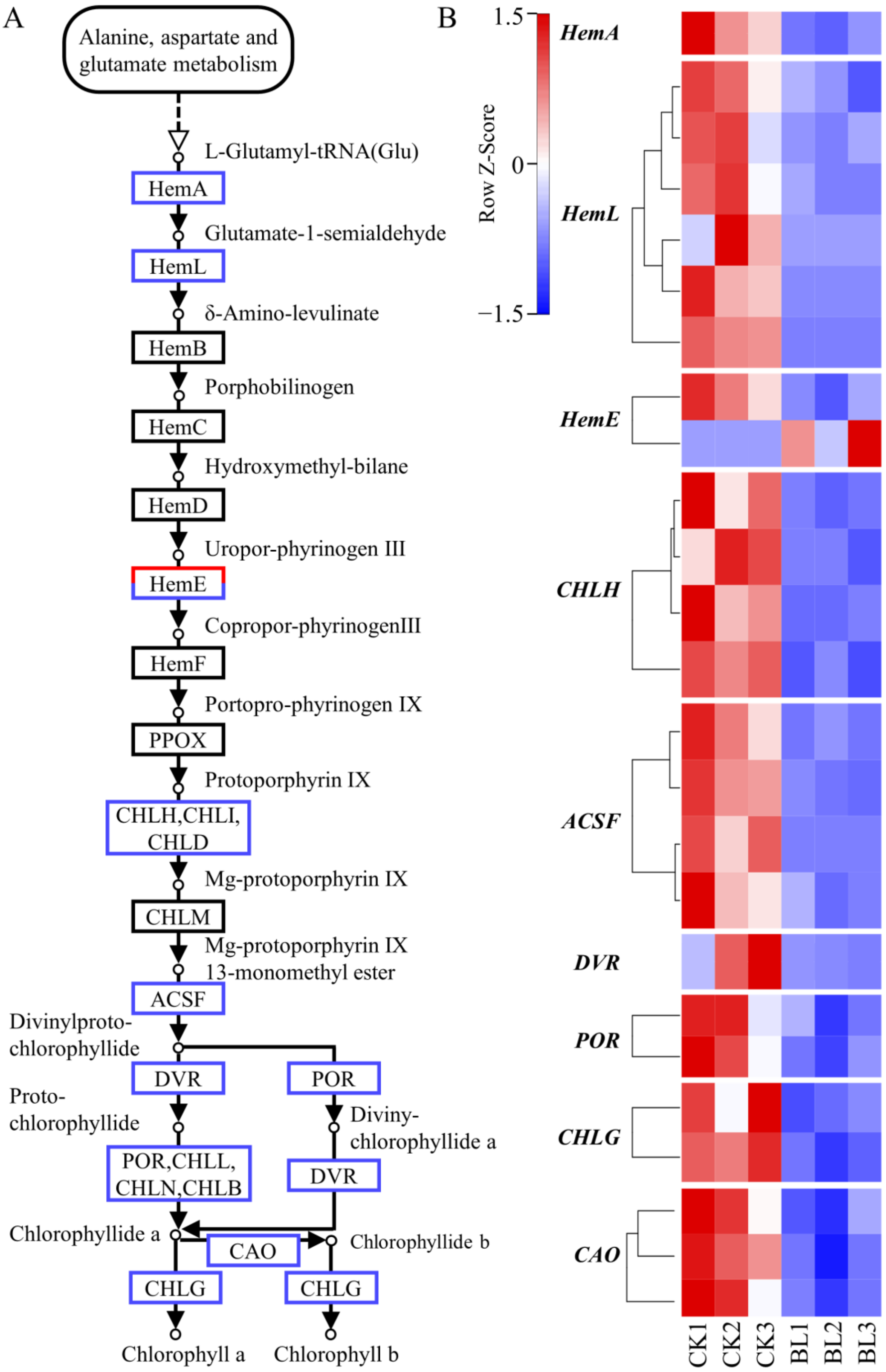

Supplementary Figure S4. Almost all of the DEGs in chlorophyll biosynthesis were down-regulated in BL treatment. **A**: Chlorophyll biosynthesis pathway (https://www.kegg.jp/entry/map00860 (accessed on 24 April 2022)); **B**: Expression heat maps of the DEGs in chlorophyll biosynthesis in the 6 samples.

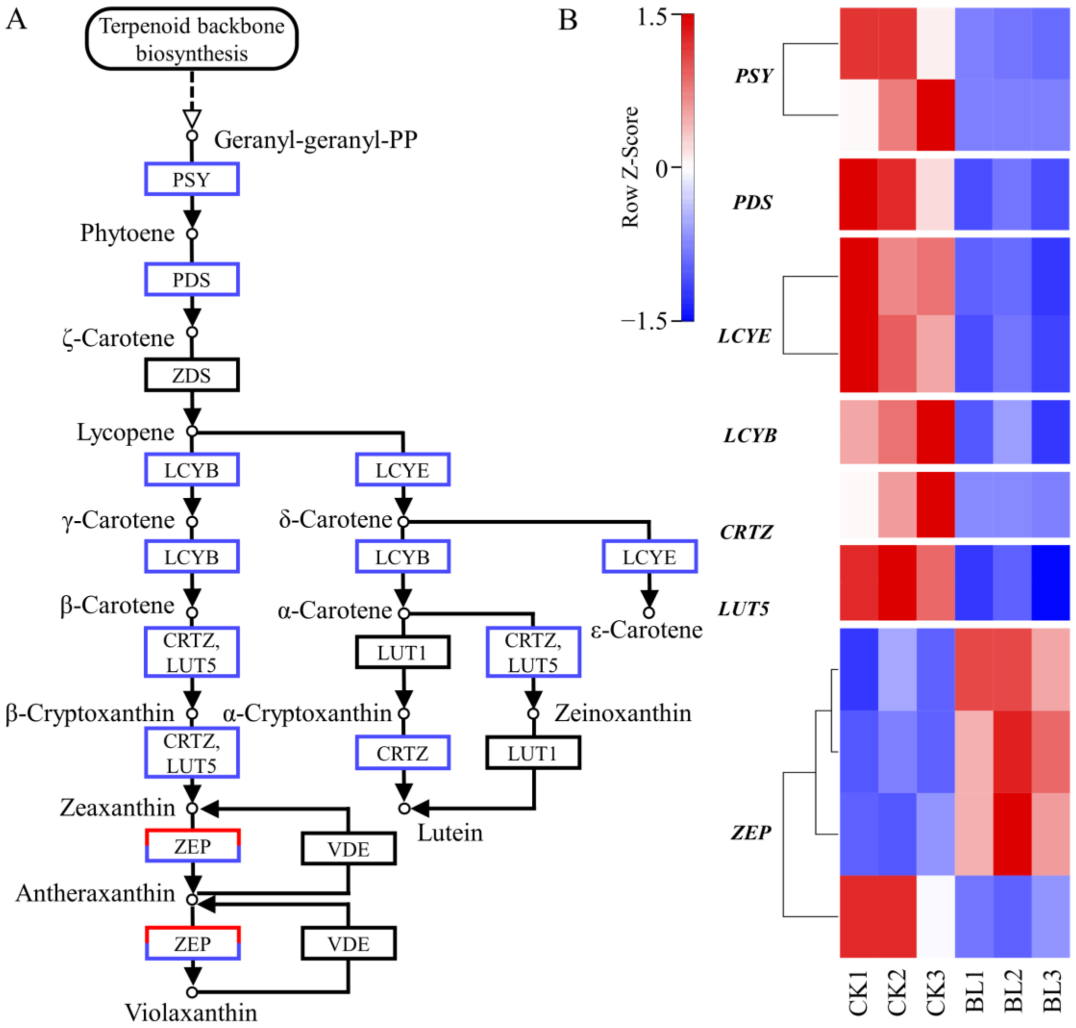

Supplementary Figure S5. The DEGs down-regulated in carotenoid biosynthesis were in the majority in BL treatment. **A**: Carotenoid biosynthesis pathway (https://www.kegg.jp/entry/map00906 (accessed on 24 April 2022)); **B**: Expression heat maps of the DEGs in carotenoid biosynthesis in the 6 samples.

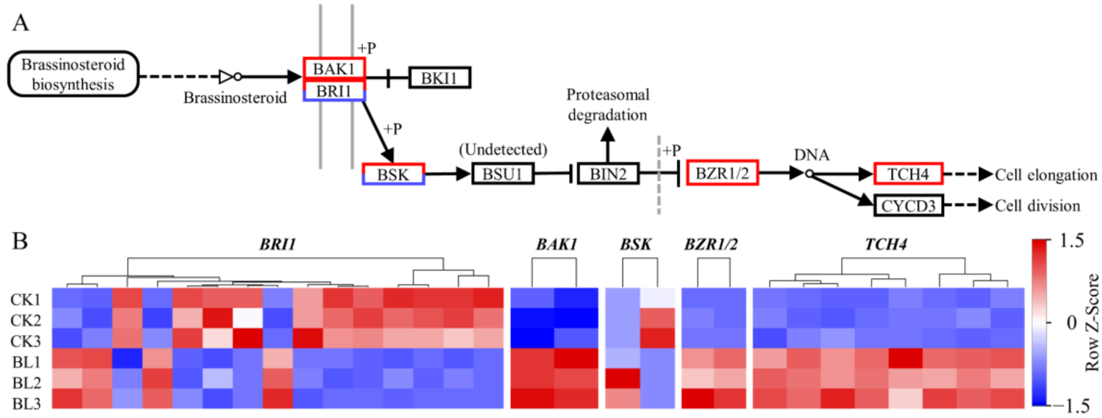

Supplementary Figure S6. Expressions of the DEGs in BR signaling. **A**: BR signaling pathway (https://www.kegg.jp/entry/map04075 (accessed on 24 April 2022)), in the pathway, *BSU1* was not detected in *A. flabellulatum* gametophytes; **B**: Expression heat maps of *BRI1*, *BAK1*, *BSK*, *BZR1/2* and *TCH4* in the 6 samples, respectively.
